# Supplementary material for: Mechanisms governing the pioneering and redistribution capabilities of the non-classical pioneer PU.1
Source: Nat Commun. 2020 Jan 21;11:402. doi: 10.1038/s41467-019-13960-2 (PMC6972792; doi:10.1038/s41467-019-13960-2)
Supplement: Supplementary file 7 — Source data [file 41467_2019_13960_MOESM7_ESM.zip › Source_Data/FigureS3/FigureS3H_MotifScanOutput/homerResults/motif1.similar.html]

motif1

## Information for motif1

C
A
G
T
A
C
G
T
G
A
C
T
C
A
G
T
A
C
T
G
G
C
A
T
A
C
T
G
C
T
A
G
A
G
C
T
G
A
C
T
C
G
A
T
A
G
T
C
  
Reverse Opposite:  

T
C
A
G
G
C
T
A
C
T
G
A
T
C
G
A
A
G
T
C
A
G
T
C
C
G
T
A
T
G
A
C
G
T
C
A
C
T
G
A
T
G
C
A
G
T
C
A
  

|  |  |
| --- | --- |
| p-value: | 1e-659 |
| log p-value: | -1.518e+03 |
| Information Content per bp: | 1.598 |
| Number of Target Sequences with motif | 1984.0 |
| Percentage of Target Sequences with motif | 51.32% |
| Number of Background Sequences with motif | 5547.7 |
| Percentage of Background Sequences with motif | 12.38% |
| Average Position of motif in Targets | 238.8 +/- 213.7bp |
| Average Position of motif in Background | 209.6 +/- 142.0bp |
| Strand Bias (log2 ratio + to - strand density) | 0.1 |
| Multiplicity (# of sites on avg that occur together) | 1.30 |
| Motif File: | file (matrix) reverse opposite |

### Similar de novo motifs found

|  |  |  |  |  |  |  |  |
| --- | --- | --- | --- | --- | --- | --- | --- |
| Rank | Match Score | Redundant Motif | P-value | log P-value | % of Targets | % of Background | Motif file |
| 1 | 0.990 | G C T A C T G A T C G A G T A C A G T C G C T A T A G C G C T A C T A G T G A C G T C A G T C A G C T A G T C A | 1e-649 | -1496.192178 | 52.66% | 13.37% | motif file (matrix) |
| 2 | 0.990 | C A G T A C G T A C G T G A C T C A G T A T C G G C A T A C T G A C T G A G C T G A C T C G A T A C G T | 1e-649 | -1494.873817 | 51.58% | 12.74% | motif file (matrix) |
| 3 | 0.970 | G C T A C T G A T C G A A G T C A G T C C G T A A G T C G T C A C T G A | 1e-614 | -1415.157267 | 52.17% | 13.83% | motif file (matrix) |
| 4 | 0.985 | G A C T C G A T C A T G G C A T C T A G A C T G A G C T A G C T C G A T A T G C G A T C | 1e-611 | -1407.920656 | 54.42% | 15.32% | motif file (matrix) |
| 5 | 0.963 | C G A T A C T G C G A T C A T G A C T G A G C T G A C T C G A T G C A T G A T C | 1e-610 | -1404.648080 | 48.65% | 11.82% | motif file (matrix) |
| 6 | 0.971 | A G C T A C T G A G C T A C T G A C T G A G C T A G C T C G A T | 1e-574 | -1322.971427 | 48.94% | 12.73% | motif file (matrix) |
| 7 | 0.951 | A G C T A C T G A C G T A C T G A C T G A C G T A C G T | 1e-532 | -1227.067393 | 40.74% | 8.98% | motif file (matrix) |
| 8 | 0.953 | G C T A G C T A T G C A A T G C A G T C C G T A A G T C T C G A | 1e-516 | -1188.249935 | 62.55% | 23.80% | motif file (matrix) |
| 9 | 0.932 | A C G T G A C T C A T G C A G T A C T G A T C G A G C T G A C T C G A T | 1e-483 | -1112.686673 | 50.28% | 15.78% | motif file (matrix) |
| 10 | 0.959 | A C G T G A T C A G C T A T C G G A C T A C T G T A C G A G C T G C A T C G A T | 1e-462 | -1065.840163 | 65.88% | 28.40% | motif file (matrix) |
| 11 | 0.913 | C A G T A C T G A C G T A C T G A C T G A C G T A G C T | 1e-376 | -867.463604 | 57.37% | 24.40% | motif file (matrix) |
| 12 | 0.862 | C G T A T A C G A G T C A G T C C T G A A G T C C G T A | 1e-367 | -845.127337 | 74.99% | 41.13% | motif file (matrix) |
| 13 | 0.889 | C G A T A G T C A C G T A C T G C G A T A C T G A T C G A C G T C G T A C T G A T C A G | 1e-345 | -795.780106 | 62.98% | 30.45% | motif file (matrix) |
| 14 | 0.836 | C G A T C A G T C A T G C G A T C T A G A T C G A G C T G A C T C G A T A G T C G A T C G C A T G C A T | 1e-193 | -445.766527 | 29.00% | 10.62% | motif file (matrix) |
| 15 | 0.822 | A C G T A C T G A C G T A C T G A C T G A T G C T G A C G C A T | 1e-191 | -441.446499 | 79.69% | 56.21% | motif file (matrix) |
| 16 | 0.777 | G A C T A G C T T C G A T G A C A G T C C T G A A G T C C G T A C T A G | 1e-107 | -248.610236 | 37.84% | 21.42% | motif file (matrix) |
| 17 | 0.781 | G T A C G C A T C G T A T C G A C T G A G T A C G A T C C G T A G A T C A T C G | 1e-105 | -243.580822 | 16.58% | 5.96% | motif file (matrix) |
| 18 | 0.789 | C A T G T G A C A C G T A C T G C G A T A C T G C T A G A T C G G C A T | 1e-63 | -145.286894 | 17.72% | 8.68% | motif file (matrix) |
| 19 | 0.796 | T A G C C A T G C G A T C A T G A C T G G A C T A G C T C G A T C T G A A T C G G T C A | 1e-28 | -65.269179 | 5.66% | 2.30% | motif file (matrix) |
